# Conformational transition induced in the aspartate:alanine antiporter by l-Ala binding

**DOI:** 10.1038/s41598-022-19974-z

**Published:** 2022-09-23

**Authors:** Satomi Suzuki, Fumika Chiba, Takuya Kimura, Nanase Kon, Kei Nanatani, Keietsu Abe

**Affiliations:** 1 Laboratory of Applied Microbiology, Department of Microbial Biotechnology, Graduate School of Agricultural Science, Tohoku University, 468-1 Aramaki-Aoba, Aoba-ku, Sendai, Miyagi 980-8572 Japan; 2 Structural Biology Group, Advanced Research Center for Innovations in Next-Generation Medicine, Tohoku University, 2-1 Seiryo-machi, Aoba-ku, Sendai, Miyagi 980-8573 Japan; 3 Microbial Genomics Laboratory, New Industry Creation Hatchery Center, Tohoku University, 6-6-10 Aramaki-Aoba, Aoba-ku, Sendai, Miyagi 980-8579 Japan

**Keywords:** Biochemistry, Microbiology

## Abstract

An aspartate:alanine antiporter (AspT) from the lactic acid bacterium *Tetragenococcus halophilus* catalyzes the electrogenic aspartate^1-^:alanine^0^ exchange reaction. Our previous kinetic analyses of transport reactions mediated by AspT in reconstituted liposomes suggested that, although the substrate transport reactions are physiologically coupled, the putative binding sites of l-aspartate (-Asp) and l-alanine (-Ala) are independently located on AspT. By using the fluorescent probe Oregon Green maleimide (OGM), which reacts specifically with cysteine, we also found that the presence of l-Asp changes the conformation of AspT. In this study, we conducted an OGM labeling assay in the presence of l-Ala. The labeling efficiency of single cysteine mutants (G62C and P79C) in transmembrane helix 3 of the AspT showed novel patterns depending on the presence of l-Ala or analogs. A concentration-dependent shift of AspT from the conformation in the presence of one substrate to that specific to the substrate added subsequently (l-Ala or l-Asp) was observed. Moreover, size-exclusion-chromatography-based thermostability assays indicated that the thermal stability of AspT in the presence of l-Ala differed from that in the presence of l-Asp. From these results, we concluded that l-Ala binding yields a conformation different from the apo or l-Asp binding conformations.

## Introduction

In the lactic acid bacterium *Tetragenococcus halophilus*, a proton-motive force (PMF) is generated by the coupled reaction of an l-aspartate decarboxylation reaction catalyzed by l-aspartate-4-decarboxylase (EC# 4.1.1.12) and an electrogenic aspartate^1-^:alanine^0^ exchange reaction catalyzed by the aspartate (Asp):alanine (Ala) antiporter (AspT), namely l-Asp (out) + l-Ala (in) → l-Asp (in) + l-Ala (out)^1,2^. The PMF is sufficiently high to drive ATP synthesis via bacterial F_0_F_1_-ATPase. This combination of PMF and ATP synthesis has been proposed as a proton-motive metabolic cycle, the prototype model of which is found in *Oxalobacter formigenes*^3–5^.

AspT is a membrane protein consisting of 543 amino acids. The membrane topology of AspT has been studied by using alkaline phosphatase and β-lactamase fusion methods^6^, as well as by the substituted-cysteine accessibility method^7^. Transmembrane helix 3 (TM3; Ile64–Met85) of AspT contains several hydrophilic amino acid residues, some of which are conserved among members of the AAEx (aspartate: alanine exchanger) family^8–11^. In our previous study, we analyzed the kinetic properties of the processes of transport of purified AspT reconstituted in liposomes and found that the putative binding sites for l-Asp and l-Ala appeared to be independently located on AspT^12^. Our finding that the l-Asp and l-Ala binding sites appear to exist independently in AspT raises the question: How does the AspT exchange reaction of l-Asp for l-Ala, and vice versa, occur? Here, we focused on l-Ala-induced conformational changes in AspT from the perspectives of the protein conformation and substrate-induced thermal stability of AspT. TM3 participates in l-Asp-induced conformational changes^10^. We performed Oregon Green maleimide (OGM) labeling assays of single cysteine mutants (G62C and P79C) in the TM3 of AspT in the presence of l-Ala. The efficiency of OGM labeling of the substituted single cysteine at TM3 depended on the concentration of the l-Ala. The l-Asp-bound conformation was shifted to the l-Ala-bound conformation by the addition of l-Ala. Next, we performed fluorescence-detection size-exclusion-chromatography-based thermostability assay (FSEC-TS) and found that the l-Ala binding conformation possessed different thermal stability profiles from the l-Asp binding conformation^13,14^. These data suggest that the binding conformations of l-Ala and l-Asp are not identical and that these conformations are mutually exclusive. Here, we discuss the conformational changes in AspT in the presence of l-Ala.

## Results

### Effect of l-Ala binding on the local configuration of AspT

**Figure 1:**
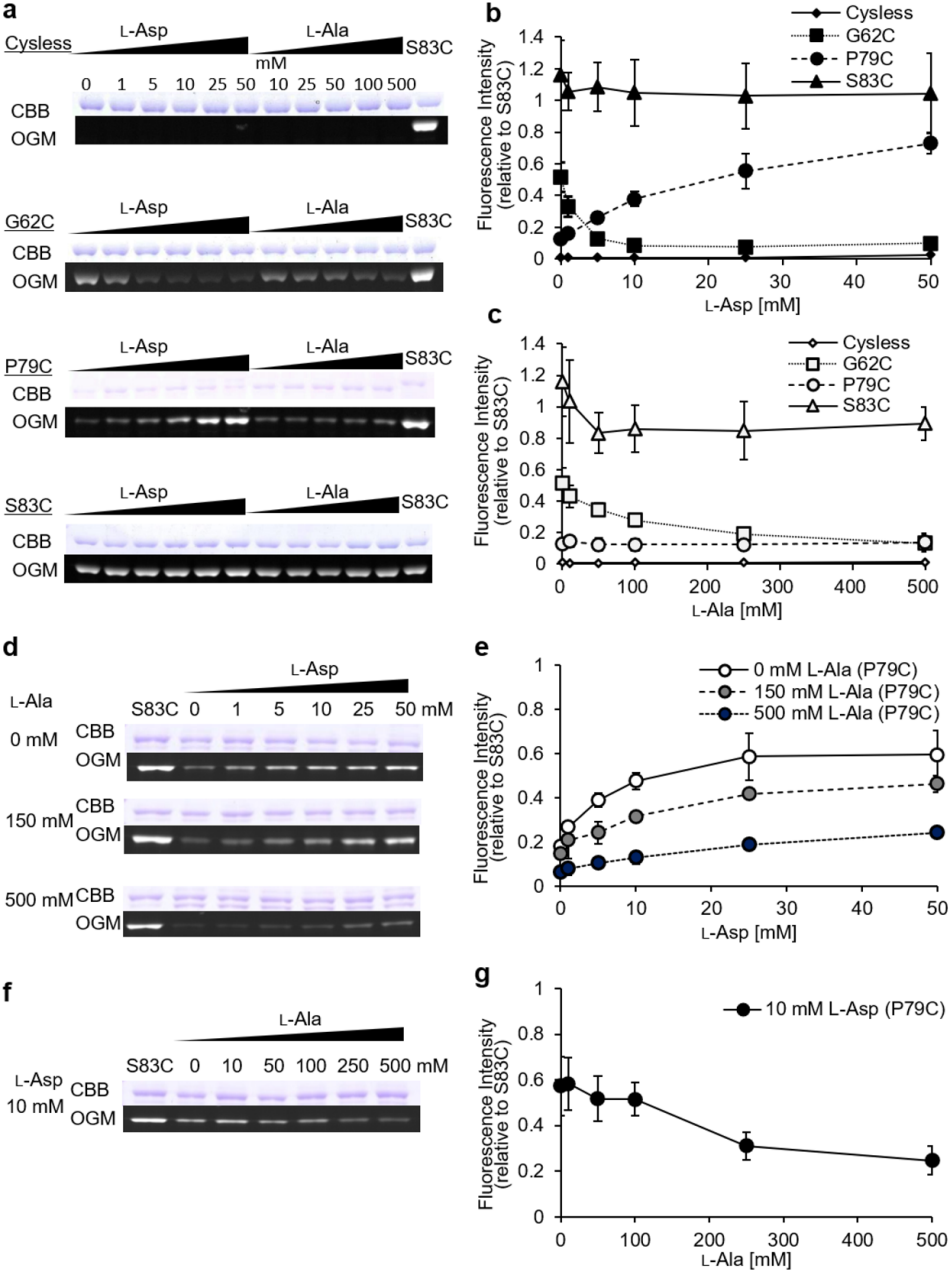
Substrate concentration dependence of OGM labelling efficiency. Membrane ghosts of each variant were exposed to OGM in the absence or presence of various concentrations of l-Asp or l-Ala before protein solubilization and purification. Purified samples were analyzed by using non-reducing SDS-PAGE (10% polyacrylamide gel matrix). (**a**) A single gel containing all of the samples was used to record the fluorescence signal with an LAS-4000 imaging system (bottom panel) before the gel was stained with CBB to visualize protein (upper panel). (**b**, **c**) Levels of fluorescence intensity and CBB staining under non-reducing SDS-PAGE were normalized against the corresponding levels of the S83C variant. The labeling efficiency of cysteine in each variant (Cysless, ♦ in **b** or ◊ in **c**; G62C, ■ in **b** or □ in **c**; P79C, ● in **b** or ○ in **c**; S83C, ▲ in **b** or Δ in **c**) is shown as a ratio of the normalized level of fluorescence intensity and CBB staining (**b**, presence of l-Asp; **c**, presence of l-Ala). (**d**–**g**) Inhibition effects of two native substrates on OGM labeling. P79C was OGM labeled under conditions in which two native substrates were present. *E. coli* cells that expressed P79C were exposed to OGM in the presence of (**d** and **e**) various concentrations of l-Asp with fixed l-Ala concentrations (0 mM, ○; 150 mM, gray dot ●; 500 mM, ●) or (**f** and **g**) various concentrations of l-Ala with fixed l-Asp concentration (10 mM, ●). The original gels are presented in Supplementary information Fig. S5.

Nanatani et al*.* previously reported that l-Asp protects a cysteine residue introduced at glycine 62 (hereinafter called G62C, Supplementary information Fig. S1) from reacting with OGM^10^. In contrast, l-Asp stimulates the reactivity with OGM of a cysteine residue introduced at proline 79 (hereinafter called P79C, Supplementary information Fig. S1)^10^. These findings demonstrate that TM3 undergoes l-Asp-induced conformational alterations. To further explore the kinetic nature of these conformational alternations, we tested the influence of the l-Ala concentration on the accessibility to OGM of the cysteine introduced at G62C and P79C (Fig. 1). S83C, which is always accessible to OGM, was used as a positive control; a cysteineless AspT variant (Cysless), which had 75% of the wild type (WT) transport activity, was used as a negative control^9^. The levels of fluorescence intensity and Coomassie Brilliant Blue (CBB) staining of the variants under non-reducing SDS-PAGE (Fig. 1a) were normalized against the corresponding levels for S83C. The labeling efficiency of cysteine in each variant is shown as the ratio of the normalized levels of fluorescence intensity and CBB staining (Fig. 1a). As shown in our previous study^10^, l-Asp stimulated the reactivity of P79C with OGM and decreased the reactivity of G62C with OGM (Fig. 1a, b). In contrast, in the presence of l-Ala the labeling patterns differed from those in the presence of l-Asp (Fig. 1a, c). l-Ala did not affect the OGM response of P79C, whereas it decreased the OGM response of G62C in a concentration-dependent manner. P79C showed no change in labeling efficiency, even at a high l-Ala concentration of 500 mM. G62C required a higher concentration of l-Ala than of l-Asp to cause a decrease in labeling efficiency.

### Competitive binding of substrate to AspT

The l-Ala-specific OGM labeling at P79C suggests that the accessibility of OGM to P79C in the presence of l-Ala differs from that in the presence of l-Asp; this may reflect the triggering of different conformational configurations by different substrates^10^. To reveal the conformation of AspT under conditions in which l-Ala and l-Asp coexisted, we further assessed OGM labeling with P79C. The extent of OGM labeling induced by l-Asp was inhibited by an increase in l-Ala concentration (Fig. 1d, e). In line with these results, l-Asp-induced OGM labeling at P79C was weakened in the presence of l-Ala at the higher concentration range (> 200 mM) (Fig. 1f, g). These data suggest that the conformation of AspT when bound with l-Asp was shifted to an l-Ala-binding conformation in response to the increase in l-Ala concentration.

### Conformational changes induced by l-Ala analog binding

**Figure 2:**
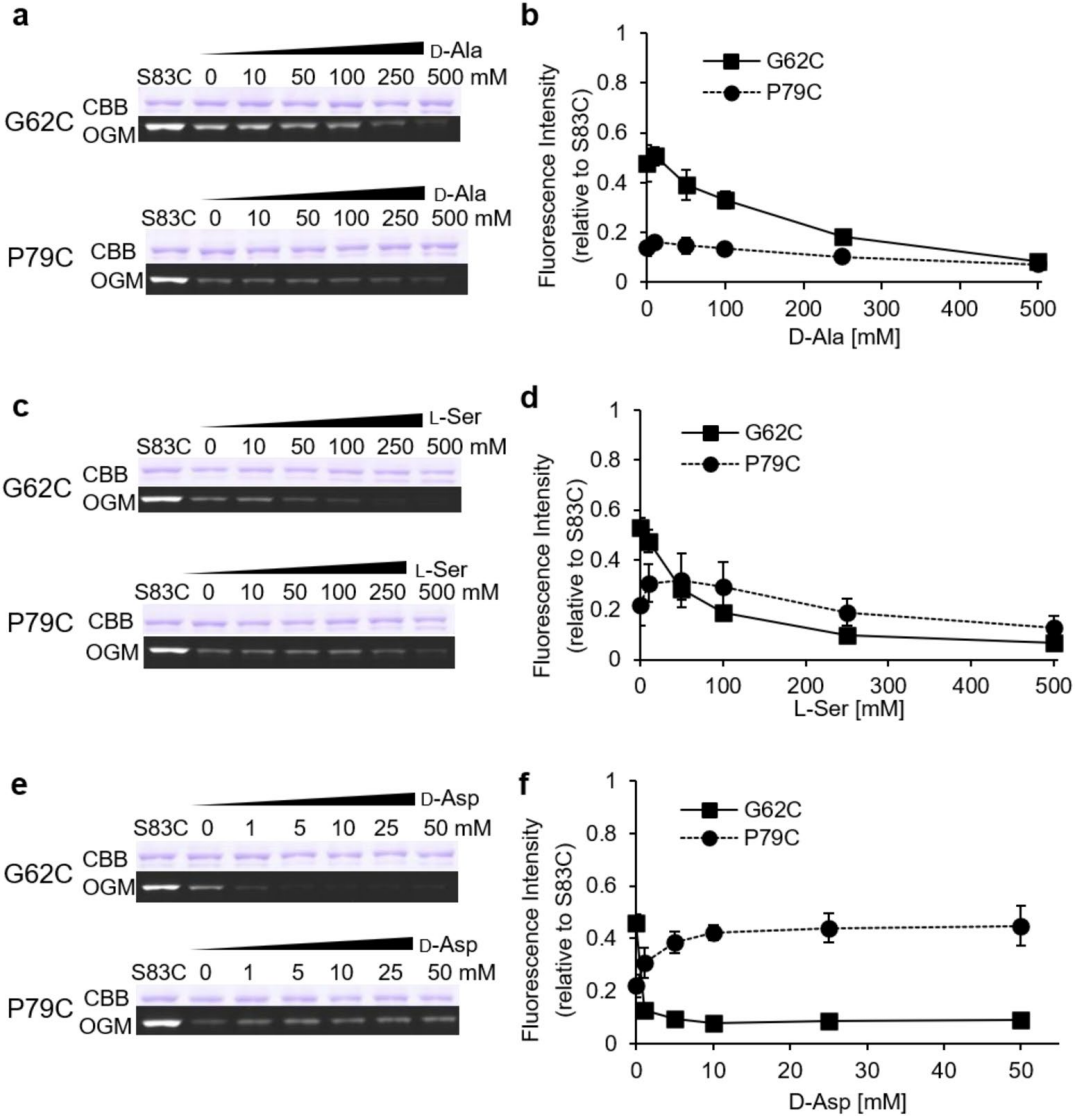
Conformational changes induced by substrate analogs. G62C (■) and P79C (●) were exposed to OGM in the absence or presence of d-Ala (**a** and **b**), l-Ser (**c** and **d**), or d-Asp (**e** and **f**). Purified proteins from OGM-exposed *E. coli* cells were analyzed by using non-reducing SDS-PAGE as described in the legend to Fig. 1. The original gels are presented in Supplementary information Fig. S6.

Previously, Sasahara et al*.* reported that the alanine analogs d-Ala and l-Ser, and the aspartate analog d-Asp, were also transported by AspT as substrates^12^. Sasahara et al. also reported that selective inhibitory effects on the self-exchange reaction of the native substrate of these analogs suggested the existence of two independent substrate-binding sites for l-Asp (and its analogs) and l-Ala (and its analogs) in AspT^12^. We confirmed that, like l-Ala or l-Asp, these analogs were transported as self-exchange substrates (Fig. S2). Then, to investigate the configurations of AspT binding to substrate analogs, we applied the OGM labeling method to G62C and P79C. In the case of the l-Ala analog d-Ala, an increase in d-Ala (Fig. 2a, b) concentration led to an apparent reduction in the OGM-labeling efficiency of G62C, as did an increase in l-Ala (Fig. 1a, c). In contrast, the labeling efficiency of P79C was not increased in the presence of d-Ala (Fig. 2a, b). In the case of the l-Ala analog l-Ser, an increase in l-Ser (Fig. 2c, d) concentration led to an apparent reduction in the labeling efficiency of G62C, as did an increase in l-Ala (Fig. 1c) and d-Ala (Fig. 2b). However, unlike in the case of d-Ala, weak stimulation of OGM labeling at P79C was observed at low l-Ser concentrations (< 50 mM) (Fig. 2b, d). Moreover, stimulated labeling gradually decreased at the higher concentrations of l-Ser (> 100 mM) (Fig. 2d). In the case of d-Asp, the OGM-labeling efficiency of G62C decreased markedly as the d-Asp concentration increased (Fig. 2e, f). In contrast, the OGM-labeling efficiency of P79C increased as the d-Asp concentration increased (Fig. 2e, f). The OGM-labeling profiles of G62C and P79C in the presence of d-Asp were in good agreement with the profiles in the presence of l-Asp (Fig. 1f). The OGM-labeling experiments at G62C and P79C in the presence of these substrates and their analogs clearly demonstrated that the OGM-labeling profiles induced by l-Ala and its analogs differed from the profiles induced by l-Asp and its analogs.

### Inhibitory nature of substrate transport reaction via AspT

**Figure 3:**
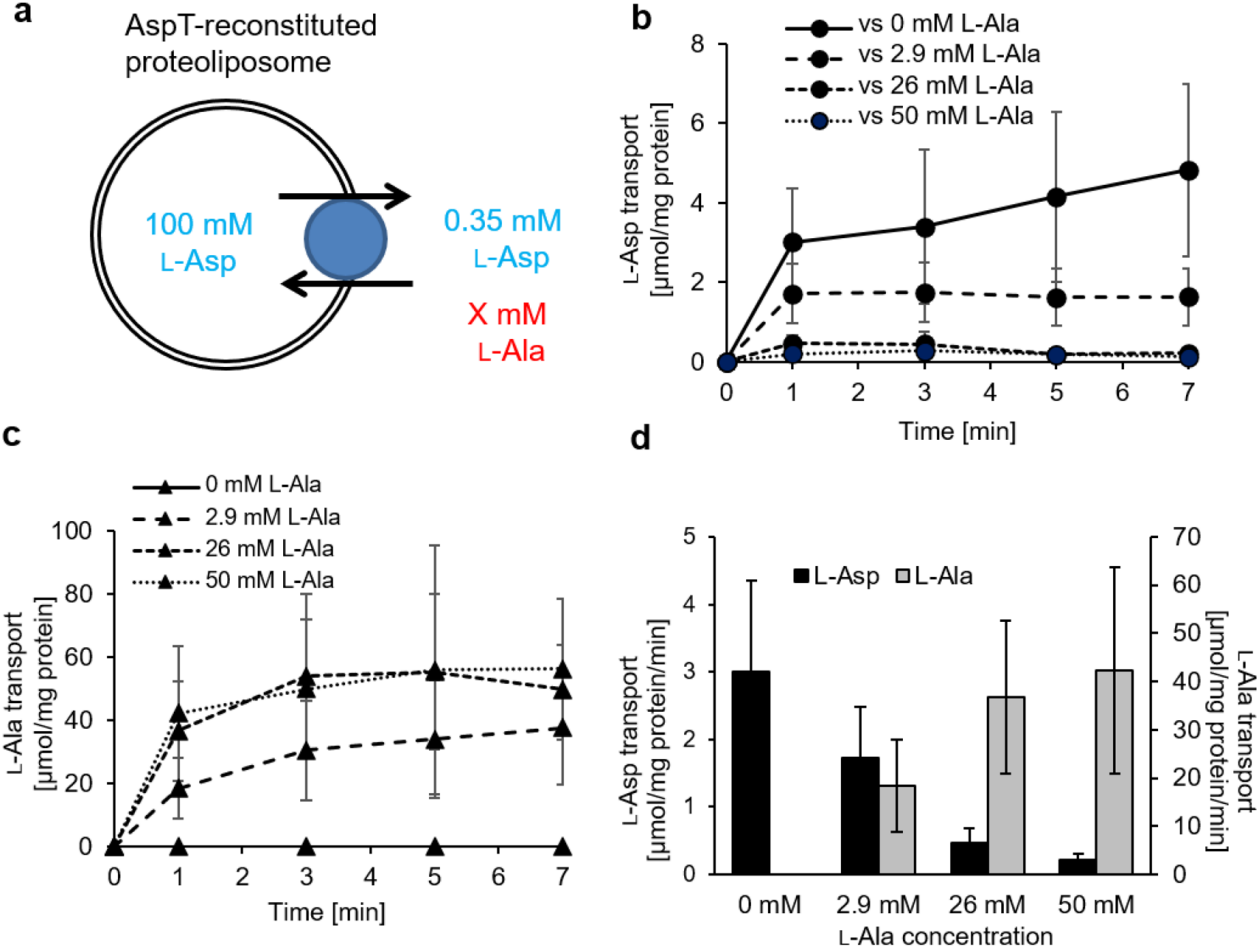
Inhibitive substrate transport assays of AspT. l-Ala and l-Asp movements were monitored by using two kinds of radioisotopes and AspT reconstituted into proteoliposomes. Proteoliposomes were loaded with 100 mM l-Asp (adjusted to pH 7 in *N*-methyl-d-glucamine [NMG]) and 50 mM potassium phosphate (pH 7) and then washed and resuspended as described in the Methods section. Proteoliposomes were placed in 50 mM NMG_2_SO_4_ plus 50 mM potassium phosphate (pH 7.0) at 5 µg of protein/mL. l-Asp at 0.35 mM [the *K*_*m*_ value] and plural l-Ala concentrations (0, 2.9 [1/10 *V*_*max*_], 26 [*K*_*m*_], or 50 mM) were added to proteoliposomes at the same time (**a**), at which point 0.5 mM l-[^14^C]aspartate (**b**) or 0.02 mM l-[^3^H]alanine (**c**) was added. To estimate substrate exchange, aliquots were taken for filtration and washing at the times indicated (0, 1, 3, 5, and 7 min). (**b** and **c**) describe counterflow, and (**d**) shows the initial rate of l-Asp (black bars) or l-Ala (gray bars) uptake into proteoliposomes. Data are from three independent experiments with three replicates per condition and are presented as averages with SD.

Because l-Ala and l-Asp showed inhibitory effects on the binding of l-Asp and l-Ala, respectively, to AspT, we considered that these substrates would inhibit the transport reaction of AspT. We therefore established a method for monitoring the transport reactions of l-Ala and l-Asp via AspT at the same time by using l-[^3^H]alanine and l-[^14^C]aspartate. For reconstitution of AspT into proteoliposomes, we followed a conventional protocol established by Sasahara et al., with the exception of the steps of phospholipid preparation (see Materials and Methods)^12,15^. We loaded 100 mM l-Asp into proteoliposomes and then added a radioactive substrate mixture (0.35 mM l-[^14^C]aspartate and 0, 2.9, 26, or 50 mM l-[^3^H]alanine) to the pre-incubated proteoliposomes. The radiolabeled substrates transported into the proteoliposomes via AspT were quantified with a liquid scintillation counter after filtration (Fig. 3a). Transport of l-Asp via AspT was inhibited by l-Ala in a concentration-dependent manner (Fig. 3b, d). In contrast, the initial rate of l-Ala uptake via AspT increased in association with the increase in l-Ala concentration (Fig. 3c, d). These data suggest that the transport of l-Ala and that of l-Asp are mutually exclusive.

### l-Ala binding induces weak thermal stability in AspT

**Figure 4:**
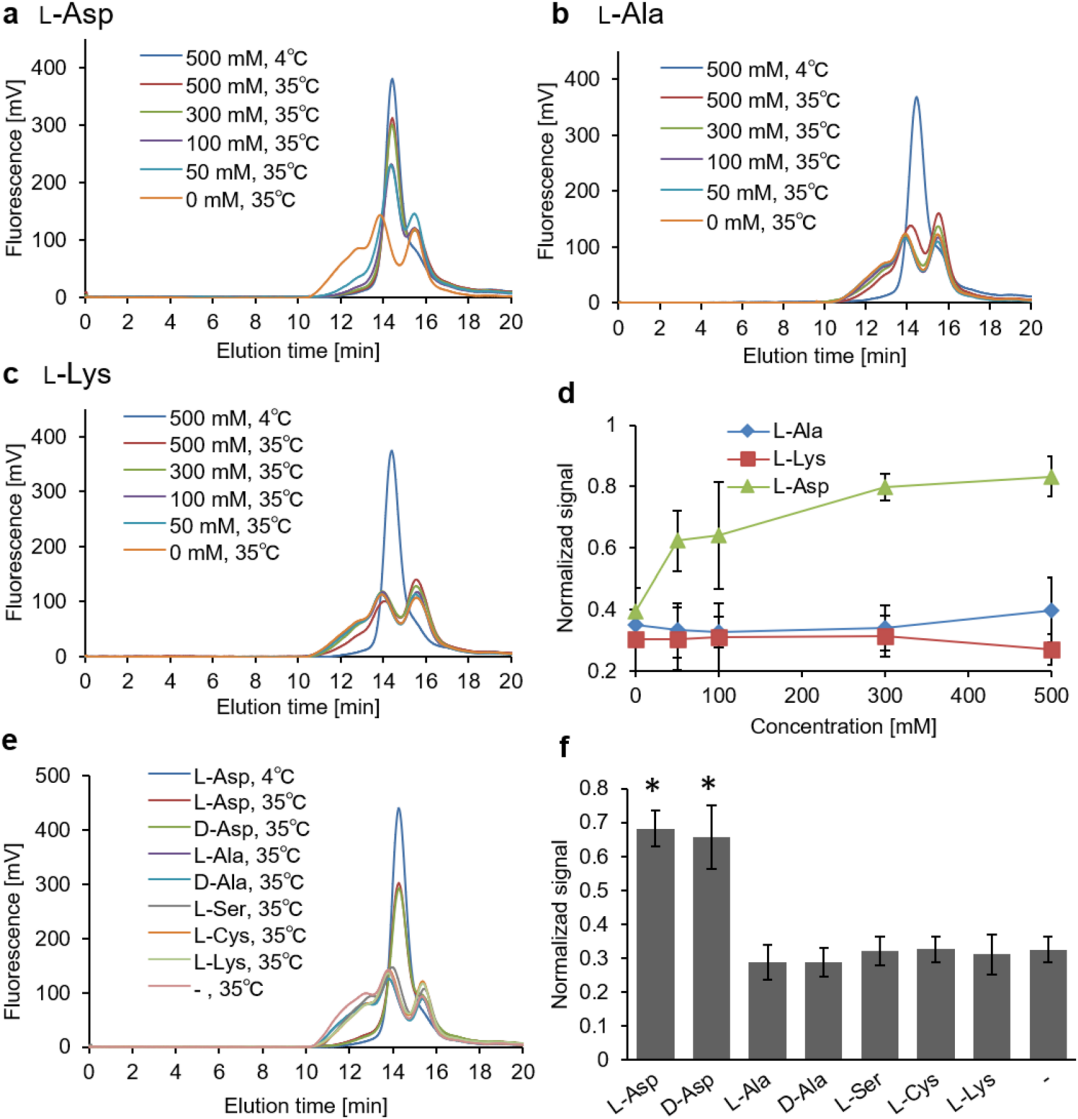
Substrate concentration dependance and its specificity for the thermal stability of AspT. Purified AspT was heat treated at 4°C or 35°C for 10 min in the presence of 0 to 500 mM of l-Asp (**a**), l-Ala (**b**), or l-Lys (**c**). After ultracentrifugation at 190,000 *g* for 30 min at 4°C to remove aggregated AspT, the supernatant was subjected to size-exclusion chromatography and the tryptophan fluorescence of AspT was observed with a fluorescence detector. The fluorescence intensities relative to that of undenatured AspT, taking the peak height of purified AspT without heat treatment as 1, are shown in (**d**). (**e**) Purified AspT was heat treated at 35°C for 10 min in the presence of 100 mM substrate or analog. The fluorescence intensities relative to that of undenatured AspT, taking the peak height of purified AspT without heat treatment as 1, are shown in (**f**). *P < 0.05, Tukey’s test.

**Figure 5:**
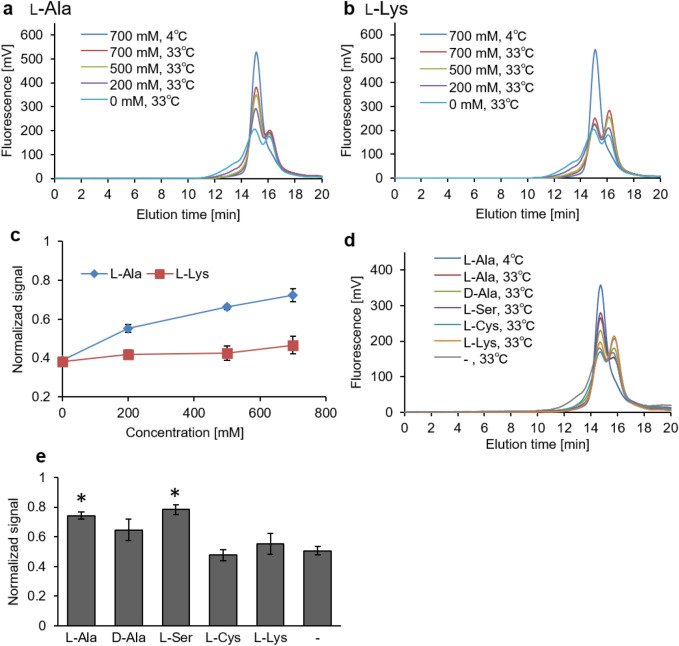
Low-affinity substrates need high concentrations to raise the thermal stability of AspT. Purified AspT was heat treated at 33°C for 10 min in the presence of 0 to 700 mM l-Ala (**a**) or l-Lys (**b**). After ultracentrifugation at 190,000 *g* for 30 min at 4°C to remove aggregated AspT, the supernatant was subjected to size-exclusion chromatography and the tryptophan fluorescence of AspT was observed with a fluorescence detector. Relative fluorescence intensities of undenatured AspT, taking the peak height of purified AspT without heat treatment as 1, are shown in (**c**). (**d**) Purified AspT was heat treated at 33°C for 10 min in the presence of 700 mM substrate or analog. Relative fluorescence intensities of undenatured AspT, taking the peak height of purified AspT without heat treatment as 1, are shown in (**e**). *P < 0.05, Tukey’s test.

To observe the substrate-concentration dependence of the thermal stability of AspT, purified AspT was diluted tenfold with buffer containing 0, 50, 100, 200, 300, or 500 mM l-Ala, l-Asp (positive control), or l-Lys (control, which was not a substrate). Constant ionic strength of each buffer was maintained by adding 0–500 mM potassium chloride. The diluted samples were then heated at 35 °C for 10 min. The thermal stability of AspT increased with increasing l-Asp concentration (Fig. 4a, d). In contrast, no effect on the thermal stability of AspT was observed in the presence of increasing concentrations of l-Ala or l-Lys (Fig. 4b–d). To investigate the effect of substrate analogs on the thermal stability of AspT, we performed FSEC-TS assays with 100 mM substrate (l-Ala or l-Asp) or substrate analogs (d-Ala, d-Asp, l-Ser, l-Cys), the *K*_m_ values of which had been previously determined by using proteoliposome reconstitution methods^12^. When l-Asp or d-Asp was present in the assay buffer, the peak of undenatured AspT was observed after heat treatment at 35°C for 10 min (Fig. 4e, f). l-Ala and the other analogs at 100 mM did not affect the thermal stability of AspT at the same temperature. We hypothesized that the reason why l-Ala—an authentic substrate of AspT—did not stabilize AspT was the low affinity of l-Ala for AspT. To observe the substrate-concentration dependence of the thermal stability of AspT in the presence of low-affinity substrates, we diluted purified AspT with buffer containing 0, 200, 500, or 700 mM l-Ala or l-Lys and incubated the mixture at 4°C or 33°C for 10 min (Fig. 5a–c). Although the low-affinity substrate l-Ala stabilized AspT in a concentration-dependent manner (Fig. 5a), non-substrate l-Lys at any concentration did not stabilize AspT (Fig. 5b, c). To investigate the effects of high-concentration analogs on the thermal stability of AspT, we exposed purified AspT samples to the same temperature (33°C) for 10 min in the presence of 700 mM substrate (l-Ala) or substrate analog (d-Ala, l-Ser, l-Cys, and l-Lys) (Fig. 5d). Not only l-Ala but also l-Ser had protective effects against heat denaturation of AspT (Fig. 5e).

## Discussion

AspT catalyzes the l-Asp:l-Ala exchange reaction: l-Asp (out) + l-Ala (in) → l-Asp (in) + l-Ala (out)^1,2^.The selective inhibitory effects of substrate analogs on Asp or Ala counterflow have been explained by the two-binding-sites model^12^. However, in living cells, l-Asp import is coupled with l-Ala export. To reveal the mechanism behind substrate binding to AspT and the events that follow this binding, we focused on the conformation of AspT. We used OGM labeling^7,10^ to detect conformational changes in AspT. A structural change in AspT induced by l-Asp binding was previously observed by monitoring the OGM labeling efficiency at G62C and P79C in TM3^10^. The labeling efficiency of substituted cysteine at G62 or P79 changes depending on an increase in the concentration of l-Asp^10^. However, l-Ala showed a different labeling pattern from that of l-Asp. Specifically, a high l-Ala concentration did not affect the OGM reaction of P79C, but it decreased the OGM reaction of G62C in a concentration-dependent manner (Fig. 1a, c). In the presence of both substrates, P79C showed a substrate-concentration-dependent shift to one of two specific labeling patterns (Fig. 1d–g). Thus, although l-Ala, like l-Asp, alters the conformation around TM3 of AspT, we found that there was a new Ala-bound conformation that differed from the Asp-bound conformation.

**Figure 6:**
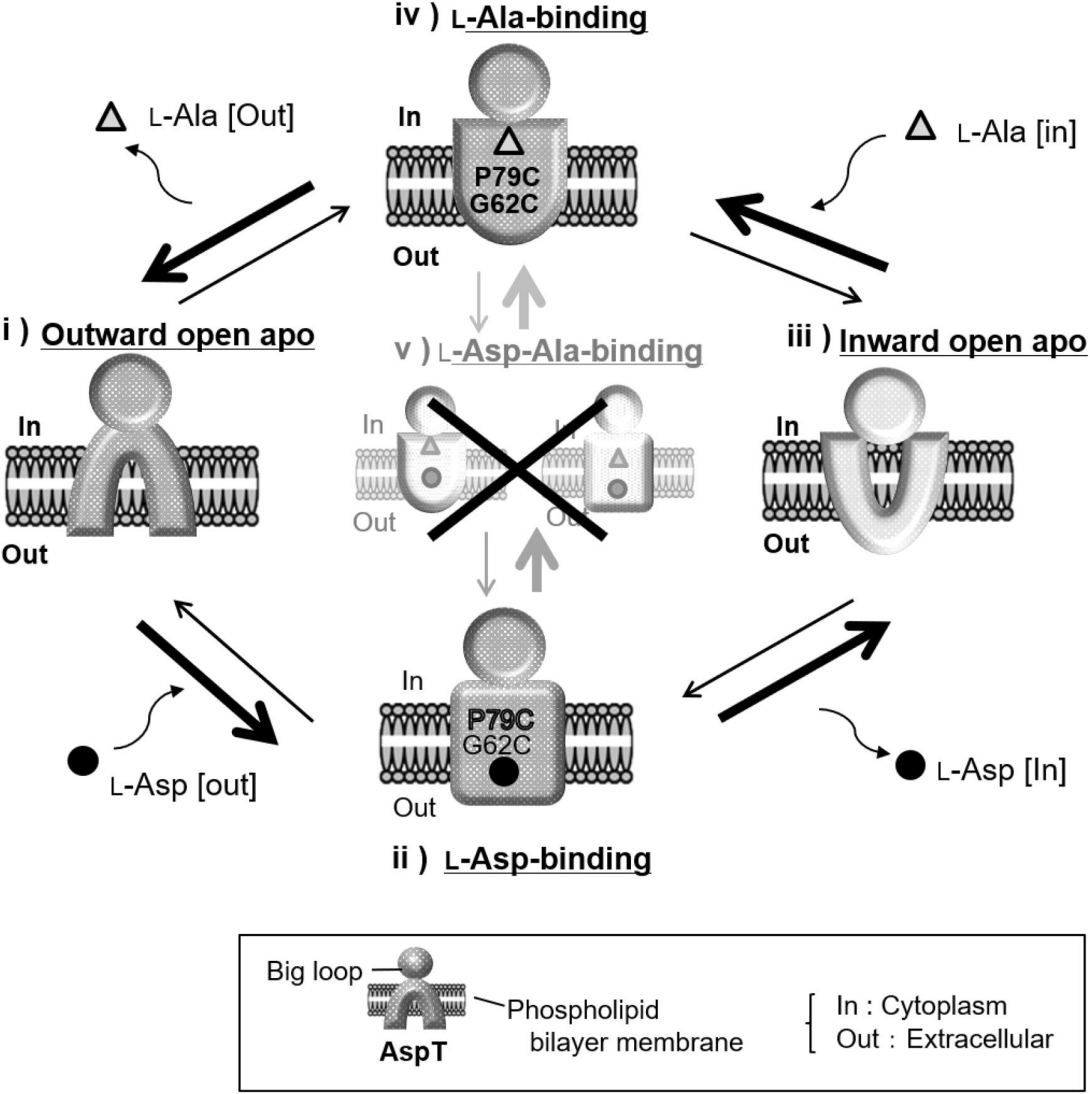
Schematic representation of the AspT substrate transport cycle. Upper is a schematic representation of the l-Ala self-exchange reaction, and Lower is a l-Asp self-exchange reaction. The outward open apo conformation changes its conformation to the l-Asp-bound conformation (ii) by an induced-fit mechanism upon binding of extracellular l-Asp (black circles; ●), and it transports l-Asp into the cell. Then AspT binds intracellular l-Ala (gray triangles; ▲), changes its conformation to an l-Ala-bound conformation by the induced-fit mechanism (iv), and releases l-Ala from the cell, thereafter returning to the outward open apo conformation (i). At present, we have no information on the conformation before l-Ala binding (iii). However, our experiments suggest that the two substrate binding forms are mutually exclusive. Therefore, there is no simultaneous substrate binding conformation (v), and after l-Asp release, intracellular l-Ala binds to AspT via the inward open apo conformation (iii), which has high binding affinity for l-Ala.

Addition of substrate analogs to the l-Ala or l-Asp counterflow reaction induced the extrusion of internal l-[^3^H]alanine or l-[^3^H]aspartate from proteoliposomes (Fig. S2), indicating that these analogs were substrates for substrate-exchange reactions catalyzed by AspT. To assess whether AspT adopts different conformations when binding to these substrate analogs, we applied OGM labeling to G62C and P79C variants. The OGM-labeling profiles induced by d-Asp (Fig. 2e, f) were similar to those induced by l-Asp (Fig. 1b). In contrast, the OGM-labeling profiles induced by d-Ala (Fig. 2a, b) and l-Ser (Fig. 2c, d) resembled those induced by l-Ala (Fig. 1c). These labeling profiles thus support the concept that l-Asp or l-Ala analogs can induce either conformation induced by l-Asp or l-Ala and can be transported via either translocation process (Fig. 6).

To assess the inhibitory behavior of the two substrates from the perspective of the conformations of AspT, we used OGM labeling assays with a P79C variant in the presence of both l-Asp and l-Ala. We found that the maximum labeling efficiencies of P79C in the presence of 50 mM l-Asp were dependent on the concentration of l-Ala present (Fig. 1d, e); the two conformations of AspT seemed to be inhibiting each other, and the abundance ratio of these two conformations depended on the concentrations of the two substrates that co-existed in the reaction mixture. These results suggest that the l-Asp or l-Ala binding conformation of AspT can be reversibly converted to the binding conformation of the other substrate.

To study the nature of l-Asp and l-Ala in inhibiting each other in the transport reaction of AspT, we established a method of chasing the transport of the two substrates (l-Asp and l-Ala) by radiolabeling with different nuclides, namely l-[^14^C]aspartate and l-[^3^H]alanine (Fig. 3b–d). A concentration-dependent inhibitory effect of l-Ala on l-Asp uptake was observed. Therefore, in the substrate transport cycle of AspT, the two endogenous substrates (l-Ala and l-Asp) have an inhibitory relationship. Taking into account the reversible conformational changes in the presence of both l-Ala and l-Asp (Fig. 1d–g), inhibition of the l-Ala or l-Asp counterflow reaction by the other substrate in the double chase assay (Fig. 3) can be attributed to inhibition between the l-Ala and l-Asp binding conformations. In other words, the binding of the two substrates of AspT (l-Asp and l-Ala) is exclusive and yields different conformations (Fig. 6, see below for details). These results suggest that l-Asp self-exchange transport and l-Ala self-exchange transport by AspT compete with each other.

Sasahara et al*.* reported that the presence of l-Asp induced thermal stability in AspT^12^. We observed the same phenomenon in size-exclusion-chromatography-based thermostability assays (Fig. 4). The increase in the thermal stability of AspT as a result of l-Asp binding can be explained as follows: Transport proteins have flexibility of function, but this flexibility generates thermal instability. In the presence of substrate, substrate binding to the transporter leads to conformational fixation and an increase in thermal stability of the transporter^16^. A similar increase in thermal stability can result from conformational regulation with site-directed point mutagenesis into transporters^14,17^. In our FSEC-TS experiment, unliganded AspT had a melting temperature of 35°C. In the presence of 100 mM l-Asp, AspT had an apparent melting temperature of 45°C, indicating that AspT in the l-Asp binding state had a 10°C increase in thermostability (Fig. S3). In contrast, 100 mM l-Ala had no effect on the thermostability of AspT (Fig. 4b), whereas 500 mM l-Ala had a stabilizing effect, giving AspT a melting temperature of 33°C (Fig. S4). The presence of l-Asp and d-Asp, which showed l-Asp binding conformation in the OGM labeling assay (Figs. 1 and 2), increased thermal stability, and the presence of l-Ala, d-Ala, or l-Ser, which showed l-Ala binding conformation (Figs. 1 and 2), led to slightly greater thermal stability than that of free AspT (Fig. 5d-e). These results indicate that the l-Asp binding conformation and the l-Ala binding conformation have different thermal stabilities, and that the l-Asp binding conformation is thermodynamically more stable than the l-Ala binding conformation. A similar phenomenon has been reported in the mitochondrial ADP/ATP carrier: A difference between specific conformations has been demonstrated by measuring the effect of mutations on the thermal stability of detergent-solubilized carriers locked in a specific state^16^. The observed difference between l-Asp concentration dependency and l-Ala concentration dependency in FSEC-TS was in good agreement with the results reported for the substrate affinity of AspT: AspT has a lower *K*_m_ value in the presence of l-Asp (0.35 mM) than of l-Ala (26 mM)^12^. Moreover, in the OGM labeling assay, l-Asp induced conformational alteration of AspT at a lower concentration than did l-Ala (Fig. 1). Overall, fixation of conformation as a result of substrate binding contributed to the increase in thermal stability of AspT in the FSEC-TS experiment. Under physiological conditions, AspT couples with AspD, an intracellular aspartate decarboxylase, to accomplish l-Asp:l-Ala conversion; AspT takes up extracellular l-Asp into the cell, and AspD decarboxylates the l-Asp into l-Ala in the cell. Therefore, *T. halophilus* has a high l-Asp concentration extracellularly and a high l-Ala concentration intracellularly; the intracellular l-Ala concentration in *T. halophilus* reaches 300 mM^1^. It can be inferred that the difference in the external l-Asp concentration and internal l-Ala concentration under physiological conditions leads to the difference in l-Asp and l-Ala concentration dependence of AspT in labeling and thermal stability experiments. We created an AspT transport model based on the above results (Fig. 6).

As shown in a previous paper, AspT has at least two conformations: a substrate-free apo conformation (Fig. 6 i) and an l-Asp-bound conformation (Fig. 6 ii). Our study revealed that there is also an l-Ala-bound conformation (Fig. 6 iv). In addition, we showed that the apo conformation of AspT has greater affinity for l-Asp than for l-Ala, suggesting that the apo AspT is an outward open apo conformation. The outward open apo conformation changes its conformation to the l-Asp-bound conformation (Fig. 6 ii) by an induced-fit mechanism upon binding of extracellular Asp (ii), and it transports l-Asp into the cell. AspT then binds intracellular l-Ala, changes its conformation to an l-Ala-bound conformation by the induced-fit mechanism (iv), and releases l-Ala from the cell, thereafter returning to the outward open apo conformation (i). At present, we have no information on the conformation before l-Ala binding. However, when Ala was added to the l-Asp-bound conformation or l-Asp was added to the l-Ala-bound conformation, AspT did not converge to the same labeling conformation (simultaneous binding of l-Asp and l-Ala); instead, AspT converged to the labeling conformation specific to the subsequently added substrate (l-Ala or l-Asp) (Fig. 1d-g). In addition, transport experiments showed a concentration-dependent shift to one of the two transport forms (Fig. 3); moreover, the thermal stability of the l-Asp-bound and l-Ala-bound forms differed (Figs. 4 and 5), suggesting that the two substrate binding forms are mutually exclusive. Therefore, there is no simultaneous substrate binding conformation (v), and after l-Asp release, intracellular l-Ala binds to AspT via the inward open apo conformation (iii), which has high binding affinity for l-Ala, resulting in the newly discovered l-Ala-binding conformation.

Symporters, such as lactose permease (LacY), are generally known to achieve simultaneous transport^18–23^. In LacY, H^+^ initially binds to the outward-facing apo conformation from outside the cell. This triggers the appearance of a specific substrate-binding site, which allows the substrate to bind. The betaine–Na^+^ symporter BetP also binds to independent substrate binding sites at the same time^24^. In contrast to symporters, in which simultaneous substrate binding and transport are common, for antiporters a different mechanism has been identified. Ca^2+^/cation (Na^+^, K^+^, H^+^, Li^+^, or Mg^2+^) antiporters export Ca^2+^ from the cell and take up cations from outside the cell. The crystal structures of some of these antiporters have been solved^25,26^. The Na^+^/Ca^2+^ exchanger (NCX_Mj) has specific binding sites for Na^+^ and Ca^2+^, but the enzymatic *K*_d_ values of Na^+^ and Ca^2+^ to NCX_Mj are different. The *K*_m_ values of both Na^+^ and Ca^2+^ are six- to eight-times lower on the cytosolic side than on the extracellular side, and the *K*_*m*_ value of Ca^2+^ is about 10 times lower than that of Na^+^^27,28^. In other words, NCX_Mj has different binding affinities and stabilities for Na^+^ and Ca^2+^ in the outward and inward states. Therefore, Na^+^ and Ca^2+^ bind, and are transported, to different specific sites at different steps of the transport cycle, and antiport is achieved without simultaneous binding. In the arginine (Arg^+^)/agmatine (Agm^2+^) antiporter AdiC, transport is thought to be achieved by the alternating binding of transport substrates to substrate-binding sites^29–32^. The same antiport mechanism has been reported for the secondary multidrug/proton antiporter MdfA^33^, the ADP/ATP carrier^34^, and the l-carnitine/γ-butyrobetaine antiporter CaiT^35,36^. Such a substrate exchange transport mechanism has been proposed as a counter-transport mechanism^37^ in the CaCA superfamily. Our findings showed that also in the case of AspT, the two substrates do not bind simultaneously but are continuously bound and released and take on two different conformations, suggesting that exchange transport is performed by a counter-transport mechanism.

To reveal the entire exchange transport mechanism of the two substrates of AspT, further studies using structural approaches (e.g., crystallographic or electron-microscopic) are necessary^38,39^. Thermodynamic and fluorescence spectroscopic studies would also be informative^36,40^.

## Methods

### Chemicals, cells, and expression plasmids

*Escherichia coli* strain XL1 blue harboring pMS421 (Spec^r^ LacI^q^) and referred to as strain XL3^3^ and strain C43(DE3) (Lucigen Corporation., Wisconsin, CA) were used to express histidine-tagged AspT with pTrc99A (Amersham Pharmacia Biotech Inc., Piscataway, NJ).

### Site-directed fluorescence labeling

Single cysteine AspT variants that retained transport activity^10^ (G62C, P79C, and S83C) and Cysless (negative control) were used to study the exposure of cysteine residue to the aqueous environment. Histidine-tagged AspT variants were expressed as described previously^10^. Membrane ghosts were prepared by using the osmotic shock method^10^ and were resuspended in 20 mM potassium phosphate buffer (pH 8) that included l-Asp or l-Ala (or both), or competitors. These membrane ghosts were incubated with these substrates for 10 min at 25°C. Then, 40 µM (final) OGM (Life Technologies, Carlsbad, CA), a membrane-impermeable thiol-active agent^33^, was added to the membrane ghosts. The mixture was incubated for 20 min at 25°C; the reaction was then quenched by the addition of 6 mM (final) β-mercaptoethanol. Membrane vesicles were harvested by centrifugation at 2900 *g* for 20 min, followed by three cycles of washing with cold distilled water and further centrifugation at 17,400* g* for 20 min. Protein was solubilized by resuspending the membrane vesicles in 1 mL of solubilization buffer (1.5% [w/v] *n*-dodecyl β-d-maltoside [DDM] in 50 mM l-Asp, 20 mM potassium phosphate [pH 7], and 20% glycerol). After incubation of the mixture at 4°C for 2 h on a rotary platform shaker, insoluble debris was removed by ultracentrifugation at 4°C (200,000 *g* for 30 min). Soluble supernatant was incubated with 50 µL of TALON metal affinity resin (Takara Bio USA, Inc., Mountain View, CA) at 4°C for 2 h. To remove nonspecific binding proteins, the resin was washed five times in wash buffer (0.01% [w/v] DDM in 50 mM l-Asp, 20 mM potassium phosphate [pH 7], and 20% glycerol). AspT was then eluted with 50 µL of elution buffer (0.01% [w/v] DDM in 50 mM l-Asp, 20 mM potassium phosphate [pH 7], 20% glycerol, and 250 mM imidazole). The elution supernatant fractions were stored at –80°C. After purification of AspT and its variants, the protein concentration of each elution fraction was determined by SDS-PAGE^11^. Labeled proteins that contained the same amount of protein as S83C (in the loading control lane) were subjected to electrophoresis. After the electrophoresis, the fluorescence profiles and CBB-stained images were recorded with a LAS-4000 imaging system (Cytiva, Tokyo, Japan) and quantified by using ImageJ^41,42^. To compare the labeling efficiencies of each sample, the OGM fluorescence intensities were divided by the CBB intensities.

### Expression, solubilization, and purification of AspT(WT)-His

For transport assays, Histidine-tagged AspT was expressed as described previously^10^. In brief, a pre-culture of *E*. *coli* XL3 carrying pTrcAspD/T-His was diluted 40-fold in fresh Luria–Bertani medium containing 30 mM d-glucose, 30 µg/mL carbenicillin, and 30 µg/mL spectinomycin. These cells were grown for 25 h at 37°C with shaking. At A_660_ (absorption at a wavelength of 660 nm) = 0.5–0.6, these cultures were diluted two-fold in the same broth containing 50 mM l-Asp and 1 mM pyridoxal 5’-phosphate. The cell suspension was then incubated statically at 37°C for approximately 3 h, and when the cultures had reached an A_660_ of 0.4 we added 200 mM isopropyl-β-d-thiogalactoside (IPTG). After an additional incubation at 37°C for 12 h, IPTG-induced cells were harvested by centrifugation at 4620 *g* for 10 min and were then washed with cold 100 mM potassium phosphate buffer (pH 7) containing 1 mM phenylmethylsulfonyl fluoride. Membrane vesicles were prepared in the same potassium phosphate buffer (pH 7) by using a high-pressure homogenizer (EmulsiFlex-B15, Avestin Inc., Ontario, Canada). Prepared membrane vesicles were solubilized^4^ at 4°C for 2 h with 1.5% (w/v) DDM in solubilization buffer (50 mM l-Asp, 20 mM potassium phosphate [pH 7], and 20% glycerol). After centrifugation of the mixture at 150,000* g* for 30 min, the supernatant was incubated with a TALON metal affinity resin (Takara Bio USA) (500 µL bed volume for a 2 L culture) at 4°C for 2 h. The column was washed at 4°C with wash buffer (50 mM l-Asp, 20 mM potassium phosphate [pH 7], 20% glycerol, 0.01% DDM) at a total of 10 mL/2 L of culture. AspT(WT)-His was then eluted by brief centrifugation at 50 *g* for 1 min with cold elution buffer (50 mM l-Asp, 20 mM potassium phosphate [pH 7], 20% glycerol, 0.01% DDM, and 250 mM imidazole) at 300 µL/2 L of culture. The elution fractions were stored at − 80°C as concentrated stocks. To determine the amount of protein in the elution fractions, 1 µL of purified protein was subjected to SDS-PAGE. It was then stained by shaking with Ultrafast Coomassie Stain (Nag Research Laboratories, Inc., Fremont, CA). The stained profiles were recorded with an LAS-4000 imaging system (GE Healthcare, Piscataway, NJ). The band intensities in the gels were quantified by using ImageJ software^41,42^.

### Reconstitution of purified AspT variants in proteoliposomes

Proteoliposome reconstitution was performed by filtering multi-lamellar vesicles (MLVs) and removing detergent by dilution^11,12,15^. First, *E. coli* phospholipid (5% w/v; Avanti Polar Lipids, Alabaster, AL) was dissolved in chloroform and sprinkled in a vortex to make a lipid film. The lipid film was then suspended in aliquots (1.1 mL) of 0.01 M potassium phosphate (pH 7.0) to produce MLVs. The MLVs (lipid film suspension) were filtered for size through a polycarbonate membrane (19 mm thick, 1 μm pore size [Avanti Polar Lipids]). Purified proteins (5 μg) were mixed with 800 μL of detergent extracts, 130 μL of filtered MLVs, and 18 μL of 15% *n*-octyl-*β*-d-glucopyranoside (Dojindo Co., Kumamoto, Japan). After the mixture had been kept for 20 min on ice, proteoliposomes (or control liposomes) were formed by rapid injection of the mixture into 20 mL of loading buffer (50 mM potassium phosphate [pH 7], plus a suitable counterflow substrate such as 100 mM l-Asp [pH 7] or l-Ala [pH 7] as the potassium salt for the l-Asp or l-Ala self-exchange reactions, respectively).

The substrate-loaded proteoliposomes (or liposomes) were harvested by ultracentrifugation at 145,250 *g* for 1 h and resuspended in assay buffer (50 mM potassium phosphate [pH 7] and 100 mM or 50 mM K_2_SO_4_ [for l-Asp or l-Ala, respectively]). To remove any outer loading buffer, the proteoliposomes (or liposomes) were washed by using the ultracentrifugation procedure just described. Finally, the proteoliposomes (or liposomes) were resuspended in 300 µL aliquots of assay buffer (50 mM potassium phosphate [pH 7], 100 mM K_2_SO_4_).

### Double chase assay of l-Asp and l-Ala

A double chase assay was used to monitor l-Asp and l-Ala transport by using two kinds of radioisotopes and proteoliposomes. For this assay, purified AspT(WT)-His was reconstituted as described above. Loading buffer (50 mM K-Pi [pH 7.0] and 100 mM l-Asp [adjusted to pH 7.0 in *N*-methyl-d-glucamine, NMG] and assay buffer (50 mM K-Pi [pH 7.0] and 50 mM NMG_2_SO_4_) were used for AspT reconstitution. Aliquots (15 µL) of proteoliposomes (or liposomes) were mixed with assay buffer (252 µL) and 1 µM valinomycin (3 µL of 100 µM valinomycin), and pre-incubated for 3 min at 25°C. Then, 0.35 mM (*K*_m_ for AspT) l-[^14^C]aspartate and plural concentrations of l-[^3^H]alanine (0, 2.9 [1/10 *V*_max_], 26 [*K*_m_ for AspT], or 50 mM) were added to the pre-incubated proteoliposomes. After substrate exchange reactions at 25°C for 1, 3, 5, and 7 min, aliquots (50 µL) of the reaction mixtures were applied to a 0.22-µm-pore-size GSTF Millipore filter (Merk KGaA, Damstadt, Germany). The membrane filters were washed twice with 3 mL of assay buffer to stop the transport reaction. After the transport assay, the proteoliposomes were subjected to SDS-PAGE for protein quantitation^11^.

### FSEC-TS assay

Purified proteins for analysis by FSEC-TS were prepared according to Miyamoto et al.^43^. To remove l-Asp and imidazole from purified AspT, purified protein was loaded onto porous polyacrylamide beads (Bio-Gel P-30 Medium, Bio-Rad). After buffer substitution, purified AspT was diluted tenfold with optional dilution buffer including substrate (l-Asp, d-Asp, l-Ala, d-Ala, and l-Ser) or other component (l-Cys and l-Lys), portioned into 80 μL aliquots, frozen in liquid nitrogen, and stored at − 80°C. For FSEC-TS assay, the sample was thawed on ice and heated at several temperatures for 10 min. To prevent additional aggregation, the heated samples were kept on ice and treated with 500 mM l-Asp and 500 mM KCl. Aggregated AspT was then removed by ultracentrifugation at 190,000 *g* for 30 min at 4°C. The supernatant was filtered through a Durapore-PVDF 0.22 µm membrane (Merk KGaA). Then, 10 μL of sample was analyzed with an HPLC system with a Superdex 200 Increase 10/300 column (Cytiva, Tokyo, Japan) in 20 mM HEPES–NaOH [pH 7] containing 0.01% DDM, 500 mM sodium chloride, and 200 mM l-Asp, with a flow rate of 0.8 mL/min. Tryptophan fluorescence (emission wavelength, 280 nm; excitation wavelength, 325 nm) of eluate was monitored by using an L-7485 fluorescence detector (Hitachi, Tokyo, Japan).

## Supplementary Information

Supplementary Information.

## Data Availability

The datasets generated and/or analyzed during the current study are not publicly available but are available from the corresponding author on reasonable request.
