# Supplementary material for: Conformational transition induced in the aspartate:alanine antiporter by l-Ala binding
Source: Sci Rep. 2022 Sep 23;12:15871. doi: 10.1038/s41598-022-19974-z (PMC9508256; doi:10.1038/s41598-022-19974-z)
Supplement: Supplementary file 1 — Supplementary Information. [file 41598_2022_19974_MOESM1_ESM.docx]

**SUPPLEMENTARY INFORMATION**

**Conformational transition induced in the aspartate:alanine antiporter by l-Ala binding**

Satomi Suzuki^1^, Fumika Chiba^1^, Takuya Kimura^1^, Nanase Kon^1^, Kei Nanatani^1,2^*^🖂^ & Keietsu Abe^1,3^*^🖂^

^1^Laboratory of Applied Microbiology, Department of Microbial Biotechnology, Graduate School of Agricultural Science, Tohoku University, 468-1 Aramaki-Aoba, Aoba-ku, Sendai, Miyagi, 980-8572, Japan. ^2^Structural Biology group, Advanced Research Center for Innovations in Next-Generation Medicine, Tohoku University, 2-1 Seiryo-machi, Aoba-ku, Sendai, Miyagi, 980-8573, Japan. ^3^Microbial Genomics Laboratory, New Industry Creation Hatchery Center, Tohoku University, 6-6-10 Aramaki-Aoba, Aoba-ku, Sendai, Miyagi, 980-8579, Japan

* Corresponding Authors

🖂Keietsu Abe, [keietsu.abe.b5@tohoku.ac.jp](mailto:keietsu.abe.b5@tohoku.ac.jp), and Kei Nanatani, [kei.nanatani.a7@tohoku.ac.jp](mailto:kei.nanatani.a7@tohoku.ac.jp), +81-22-757-4355

**SUPPLEMENTARY MATERIALS & METHODS**

**Substrate/analog exchange assay.** For the exchange reaction, purified AspT(WT)-His was reconstituted as described in the Methods. Loading buffer (50 mM K-Pi [pH 7.0] and 50 mM l-Asp [pH 7.0] or l-Ala [pH 7.0]) and assay buffer (50 mM K-Pi [pH 7.0] and 50 mM K_2_SO_4_) were used for AspT reconstitution. Aliquots (25 µL) of proteoliposomes (or liposomes) were mixed with assay buffer and preincubated for 3 min at 25°C. The radioactive substrate (l-[^3^H]alanine or l-[^3^H]aspartate) and non-radioactive substrate (100 µM l-Ala or 100 µM l-Asp) mixtures were added to the pre-incubated proteoliposomes. After substrate uptake reactions at 25°C for 1, 5, 10, and 20 min for l-Ala uptake or 1, 3, 5, and 7 min for l-Asp uptake, aliquots (50 µL) of the reaction mixtures were applied to a 0.22-µm-pore-size GSTF Millipore filter (Merck KGaA, Damstadt, Germany). The membrane filters were washed twice with 3 mL of assay buffer to stop the transport reaction. Furthermore, after 20 min (for the l-Ala exchange reaction) or 7 min (for the l-Asp exchange reaction) counterflow reactions, we added l-Ala, d-Ala, or l-Ser (for the l-Ala exchange reaction) or l-Asp or d-Asp (for the l-Asp exchange reaction) to a final concentration of 50 mM. We then removed aliquots (55.6 µL) and stopped the reaction at 20.5, 21, and 22 min (for the l-Ala exchange reaction),or 7.5, 8, and 9 min (for the l-Asp exchange reaction). After the exchange reactions, we quantified the reconstituted protein in the proteoliposomes, as described in the Methods.


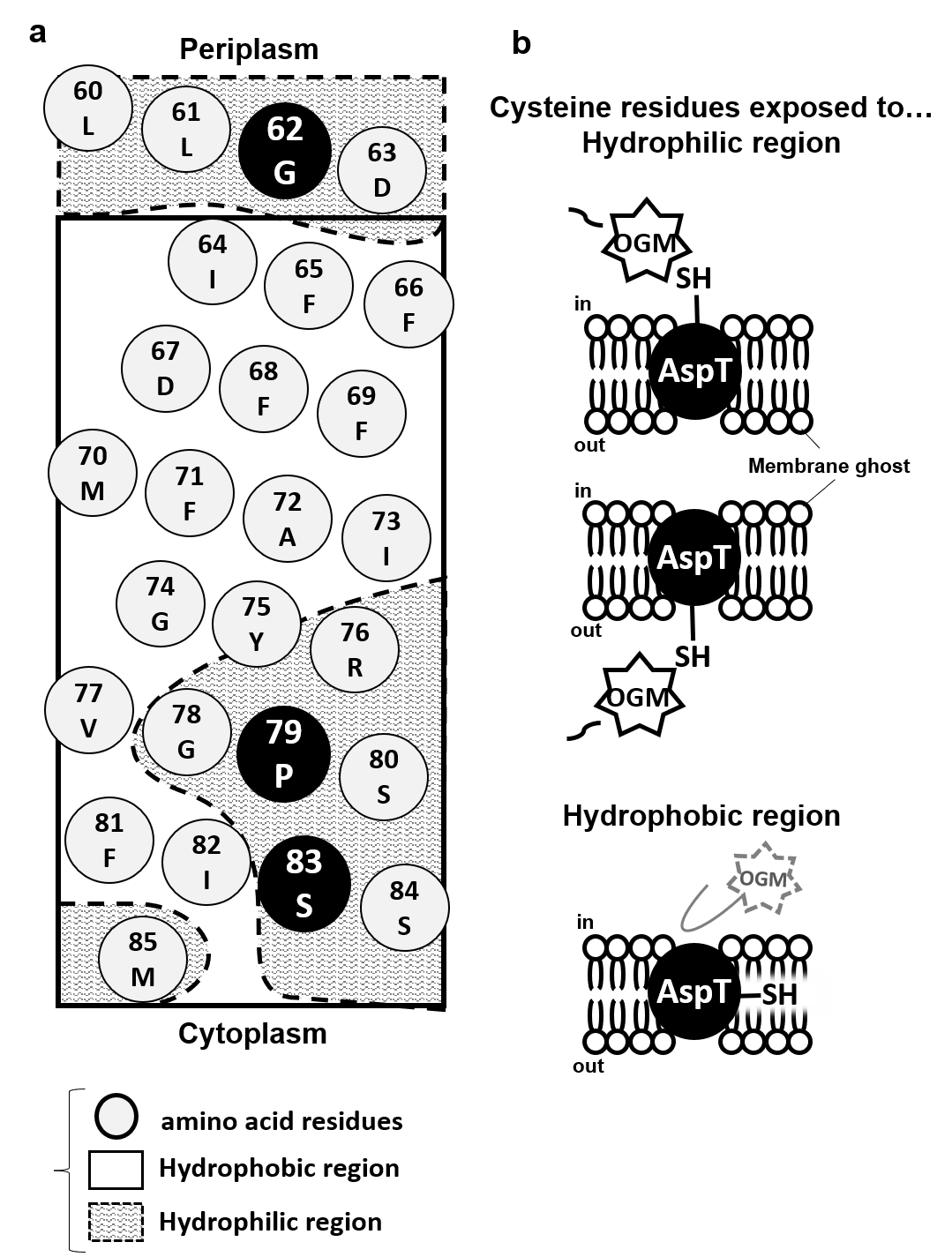
**SUPPLEMENTARY FIGURES**

**Fig. S1 Putative topological model of TM3 and schematic representation of site-directed fluorescence labeling.** Side view of TM3 from cysteine-scanning mutagenesis (**a**) (10). The amino acid residues analyzed in our experiment (G62, P79, and S83) are indicated by black circles. Previous studies have reported that each is located in the hydrophilic region and that Oregon Green maleimide (OGM) labeling efficiency changes in the presence of L-Asp (10). Because the OGM labeling experiment used membrane ghosts, the cysteine residues were labeled from both sides of the membrane and the orientation of the cysteine residues had no effect on the results (**b**).

**
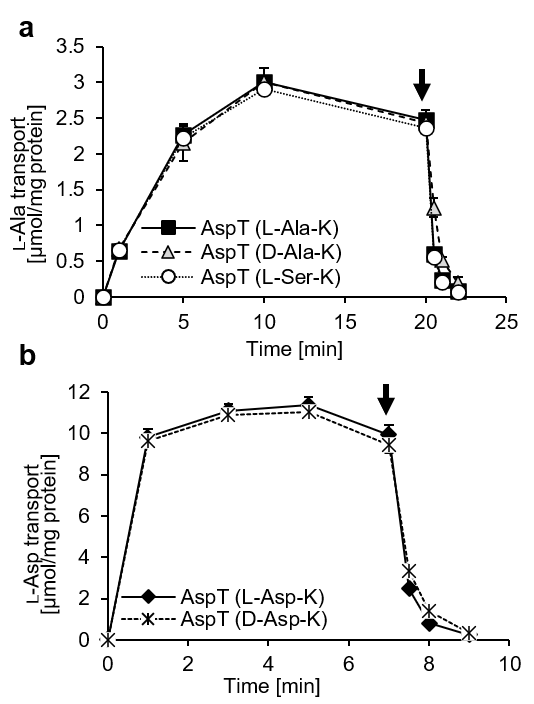
**

**Fig. S2** **Substrate/analog exchange reaction of AspT.** Substrate and inhibitor exchange reactions using AspT-reconstituted proteoliposomes. Proteoliposomes were loaded with 50 mM l-Ala (**a**) or l-Asp (**b**) plus 50 mM potassium phosphate (pH 7) and then washed and resuspended as described in the Methods. Proteoliposomes were placed in 50 mM K_2_SO_4_ plus 50 mM potassium phosphate (pH 7) at 10 µg of protein/mL, at which point 0.017 mM l-[^3^H]alanine (**a**) or 0.025 mM l-[^3^H]aspartate (**b**) was added. To estimate substrate transport, aliquots were taken for filtration and washing at the times indicated. Arrow denotes the addition of buffer, 50 mM unlabeled l-Ala (■), d-Ala (Δ), and l-Ser (○), or l-Asp (♦) and d-Asp (*).


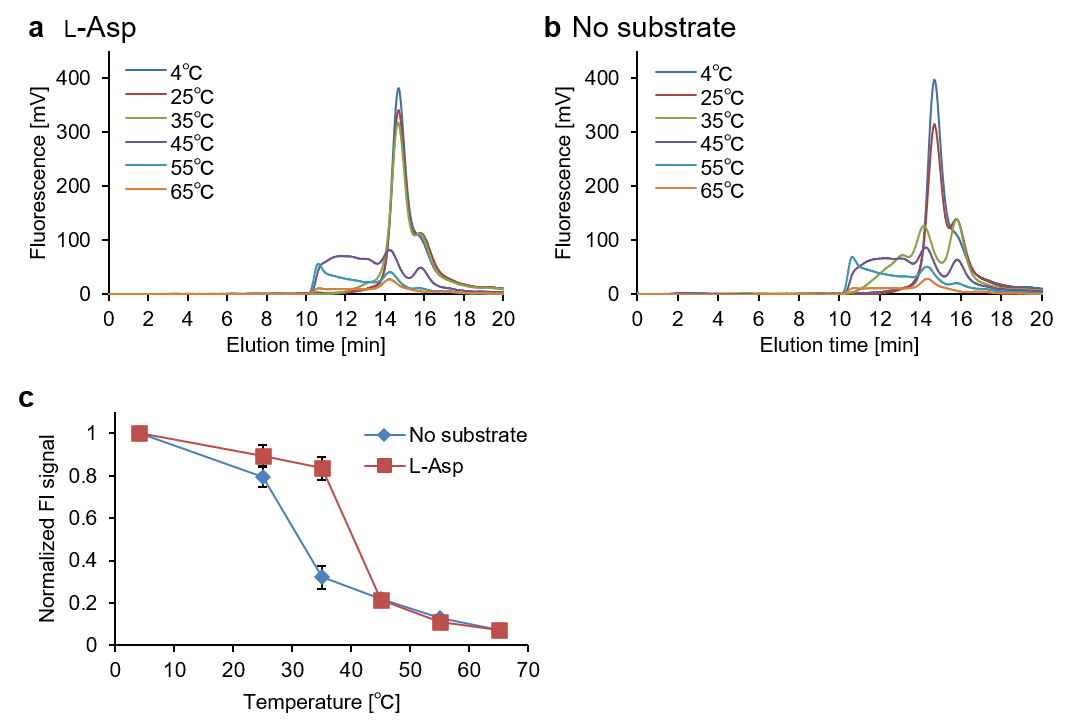


**Fig. S3 l-Asp binding induces thermal stability.** Purified AspT was heat treated at 4 to 65°C for 10 min in the presence (**a**) or absence (**b**) of 100 mM l-Asp. After ultracentrifugation at 190,000 *g* for 30 min at 4ºC to remove aggregated AspT, the supernatant was subjected to size-exclusion chromatography and the tryptophan fluorescence of AspT was observed with a fluorescence detector. The fluorescence intensities relative to that of undenatured AspT, taking the peak height of purified AspT without heat treatment as 1, are shown in (**c**). FI, fluorescence intensity


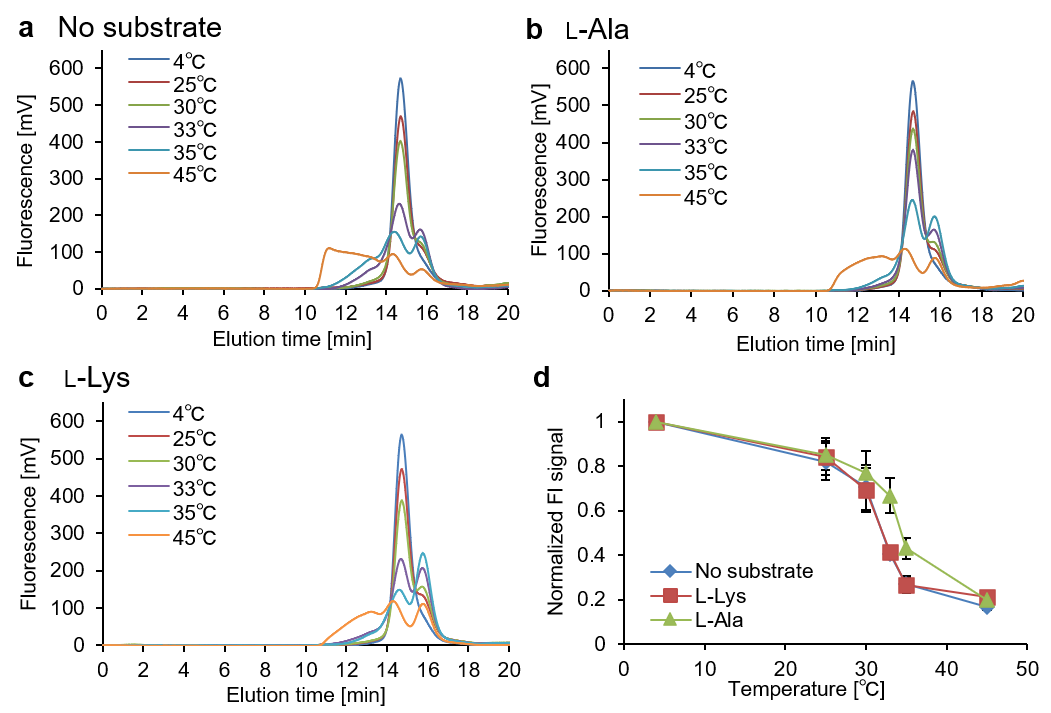


**Fig. S4** **Screening of appropriate heat-treatment temperature for low-affinity substrate.** Purified AspT was heat treated at 4 to 45°C for 10 min without substrate (**a**) or in the presence of 500 mM l-Ala (**b**) or l-Lys (**c**). After ultracentrifugation at 190,000 *g* for 30 min at 4ºC to remove aggregated AspT, the supernatant was subjected to size-exclusion chromatography and the tryptophan fluorescence of AspT was observed with a fluorescence detector. Fluorescence intensities relative to that of undenatured AspT, taking the peak height of purified AspT without heat treatment as 1, are shown in (**d**). FI, fluorescence intensity


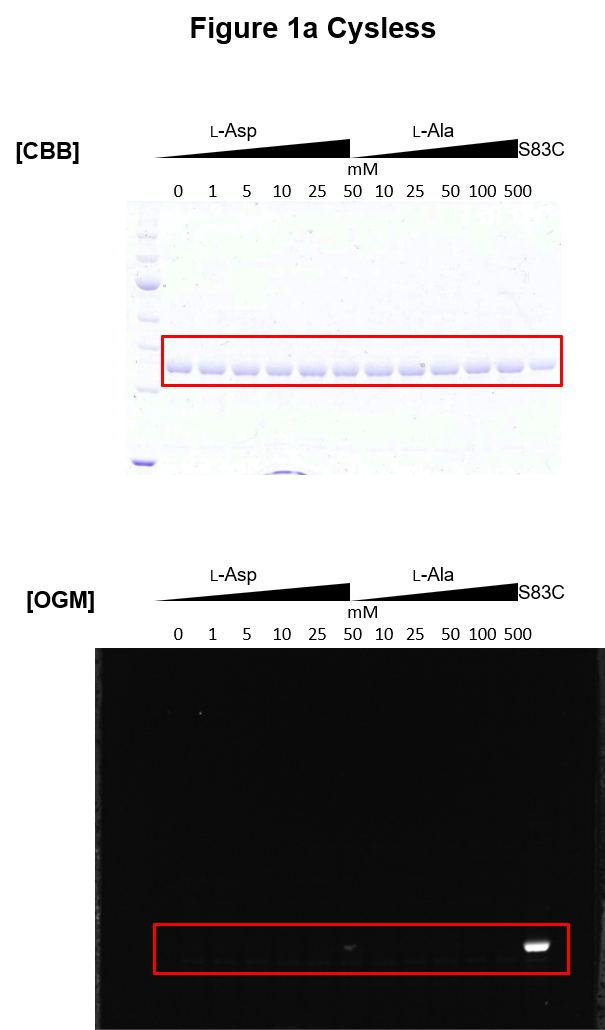


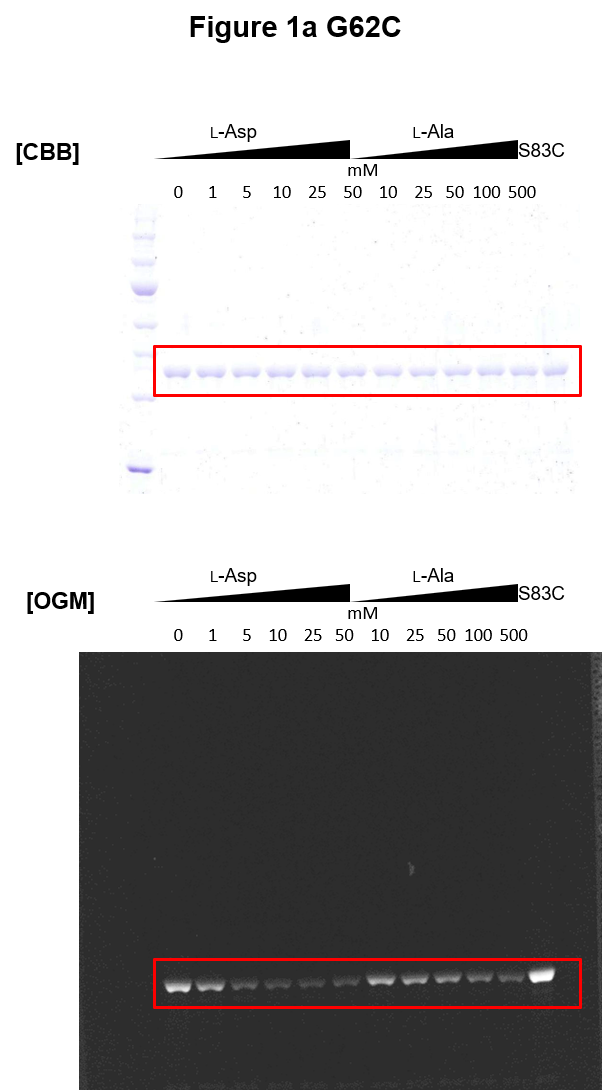


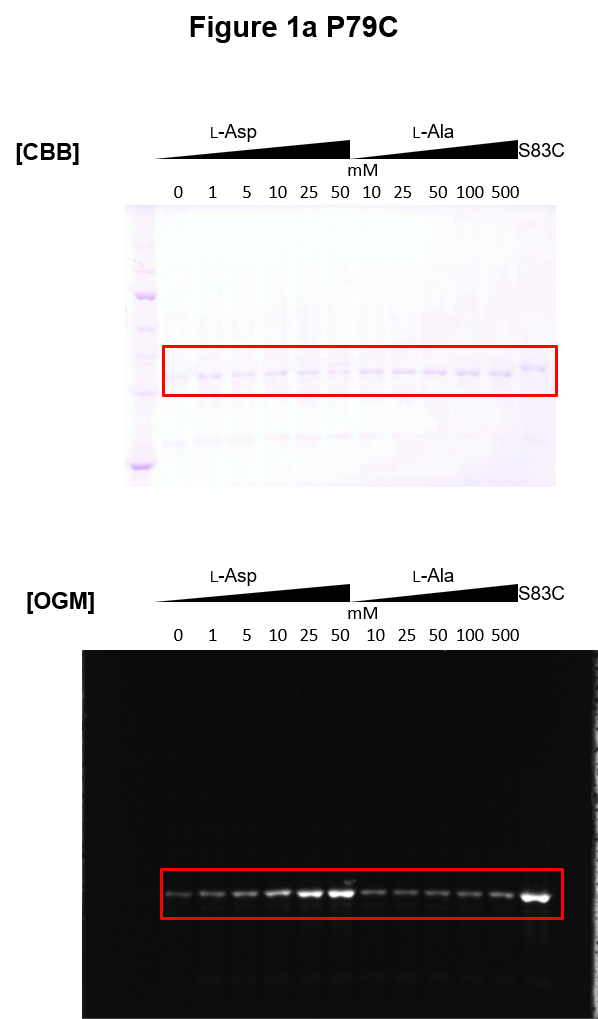


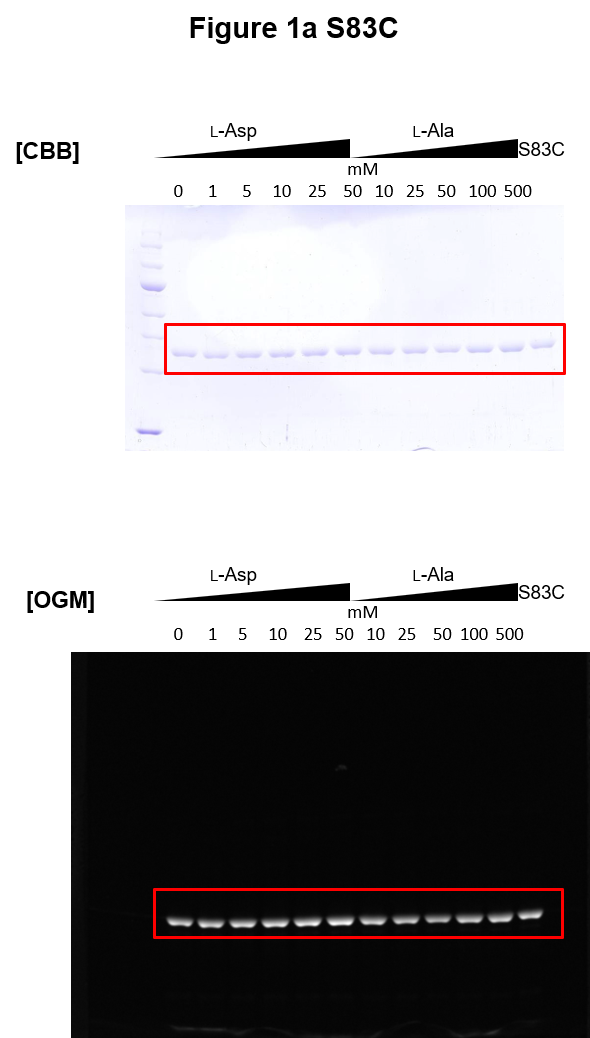


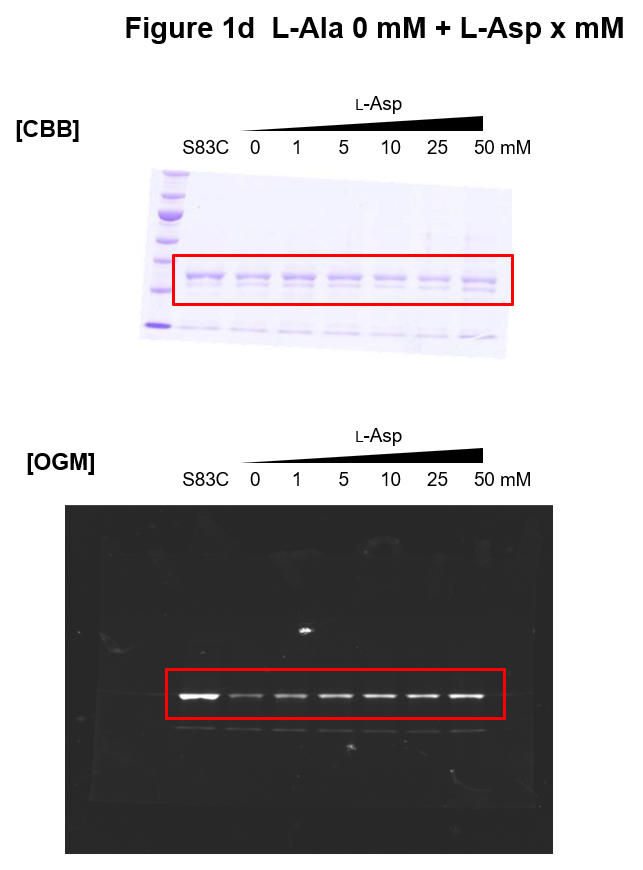


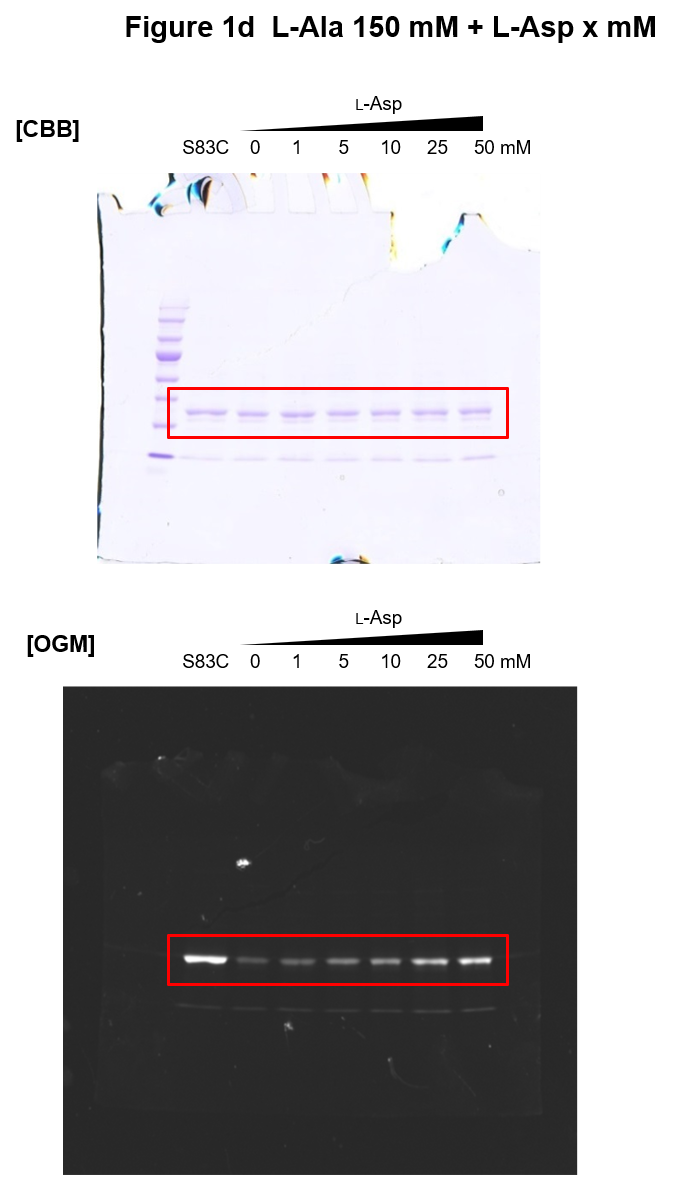


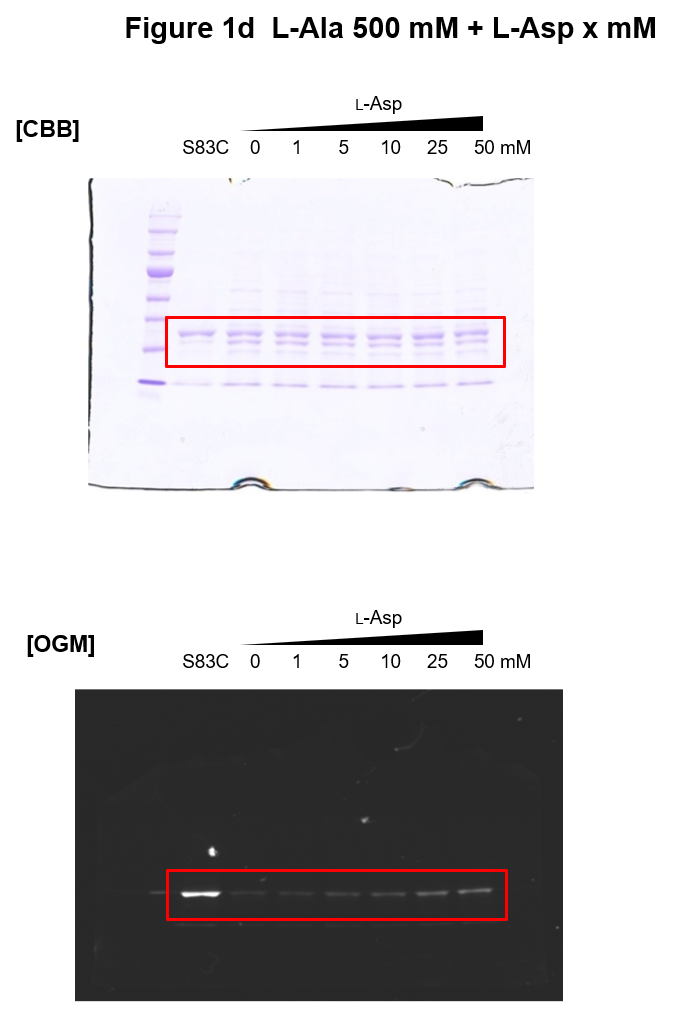


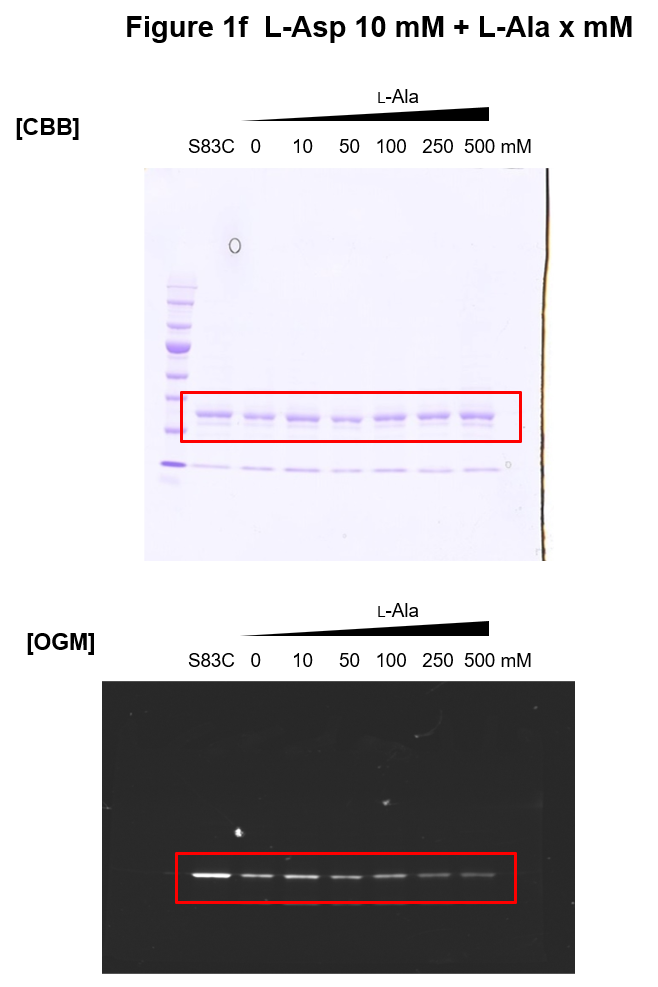


**Fig. S5 Uncropped CBB stained gels and OGM fluorescence signal for Fig 1a, d, and f.**

The original CBB stained gels and OGM fluorescence signal for the main figures are shown. The red frames indicate the bands that are shown in the corresponding figures.


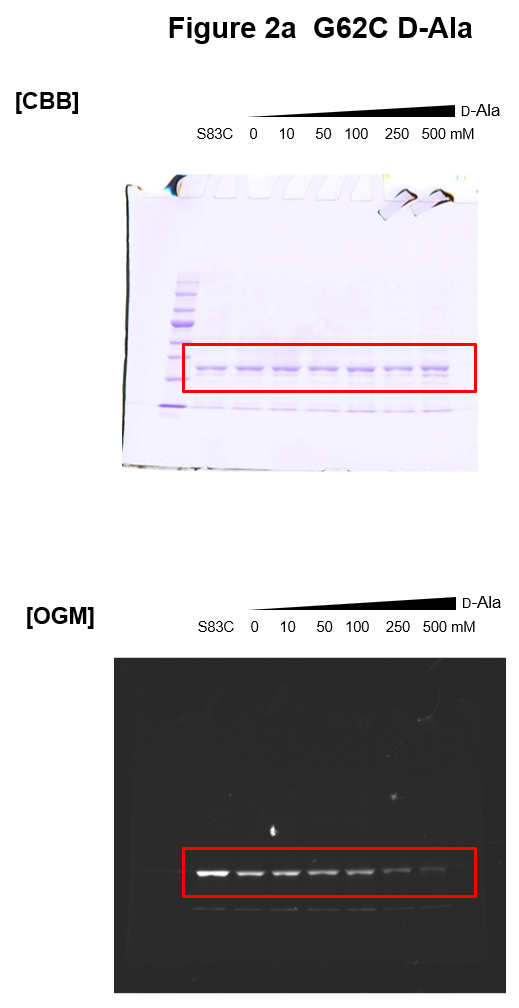


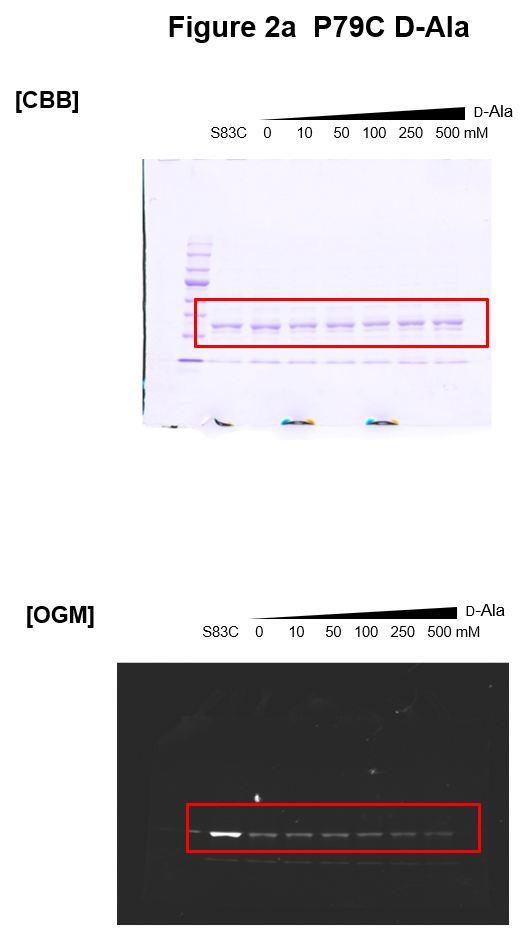


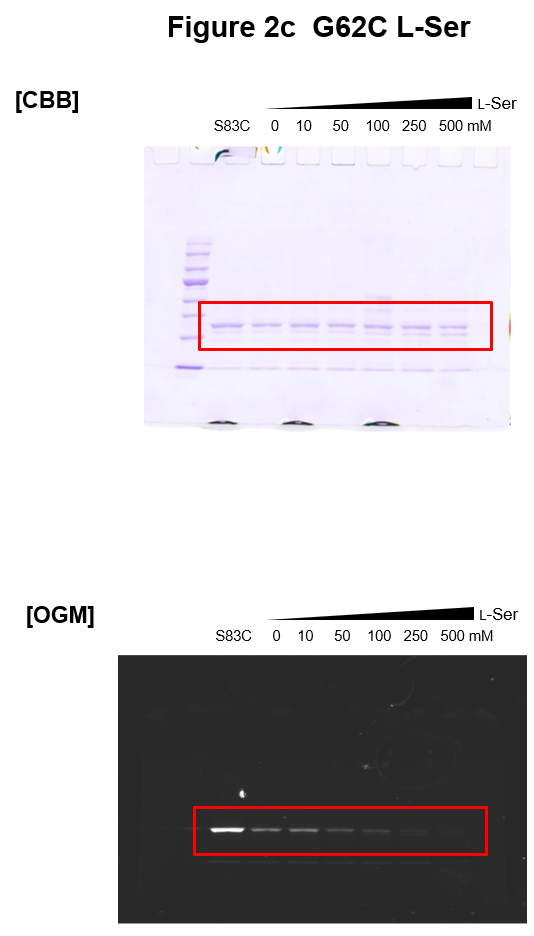


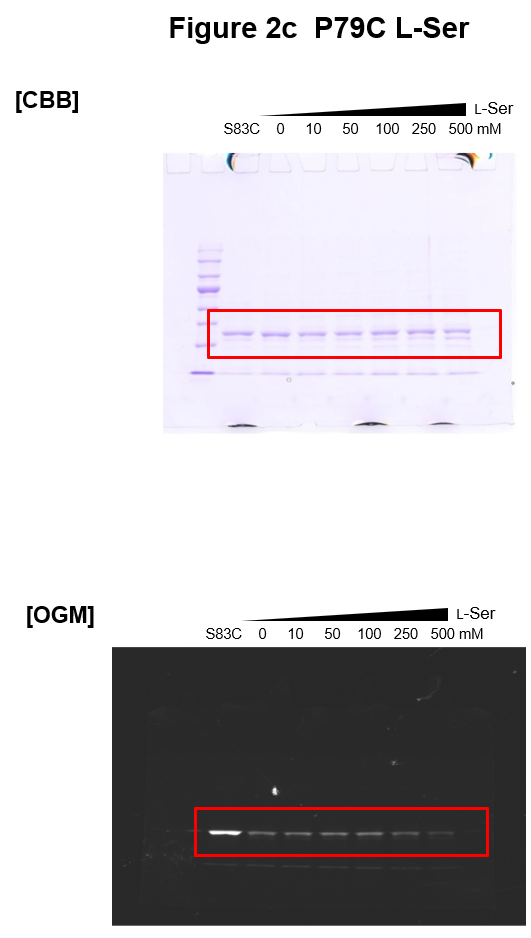


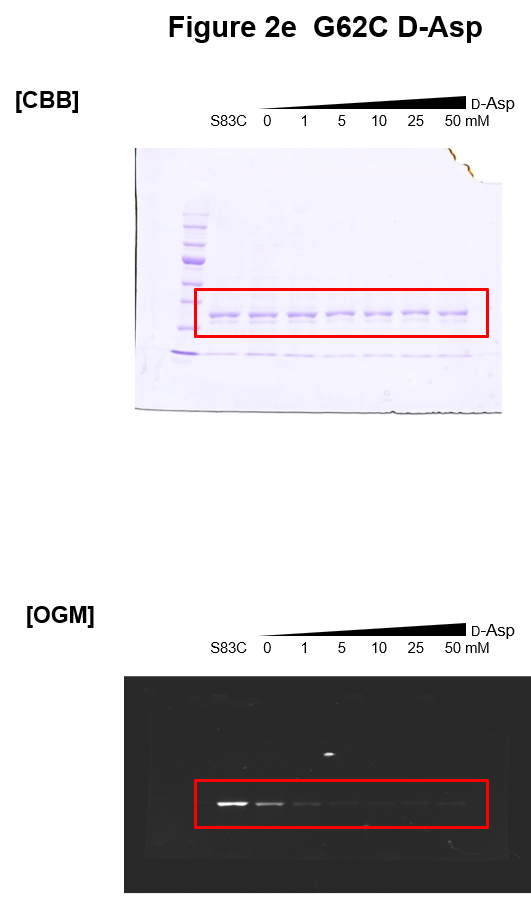


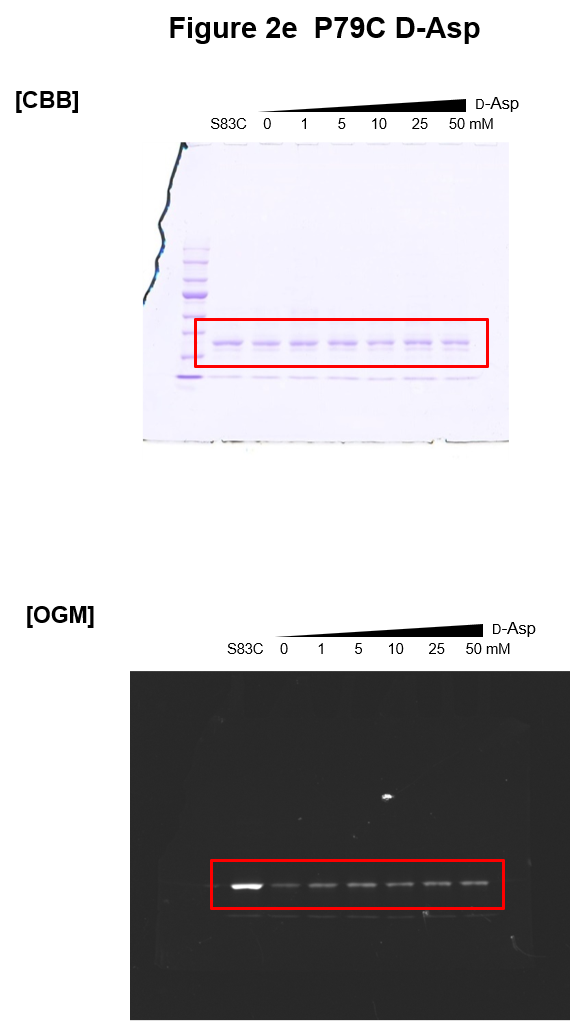


**Fig. S6 Uncropped CBB stained gels and OGM fluorescence signal for Fig 2a, c, and e.**

The original CBB stained gels and OGM fluorescence signal for the main figures are shown. The red frames indicate the bands that are shown in the corresponding figures.
